# Quality of intervention delivery in a cluster randomised controlled trial: a qualitative observational study with lessons for fidelity

**DOI:** 10.1186/s13063-017-2189-8

**Published:** 2017-11-17

**Authors:** Karen James, Alan Quirk, Sue Patterson, Geoff Brennan, Duncan Stewart

**Affiliations:** 1 Kingston University and St George’s University of London, Joint Faculty of Health, Social Care and Education, Grosvenor Wing, Cranmer Terrace, London, SW17 ORE UK; 2 Royal College of Psychiatrists, College Centre for Quality Improvement, 21 Prescot Street, London, E1 8BB UK; 3 Metro North Mental Health and Griffith University, Herston, QLD 4029 Australia; 4 Royal Brisbane and Women’s Hospital, Herston, QLD 4029 Australia; 5 Star Wards, 63 Highgate High Street, London, N6 5JX UK; 6 Mental Health and Addiction Research Group, University of York, ARRC Building, University of York, Heslington, York, YO10 5DD UK

**Keywords:** Randomised controlled trial, Safewards, Mental health nursing, Process evaluation, Implementation, Inpatient, Qualitative, Participant observation, Fidelity, Participant responsiveness

## Abstract

**Background:**

Understanding intervention fidelity is an essential part of the evaluation of complex interventions because fidelity not only affects the validity of trial findings, but also because studies of fidelity can be used to identify barriers and facilitators to successful implementation, and so provide important information about factors likely to impact the uptake of the intervention into clinical practice. Participant observation methods have been identified as being particularly valuable in studies of fidelity, yet are rarely used. This study aimed to use these methods to explore the quality of implementation of a complex intervention (Safewards) on mental health wards during a cluster randomised controlled trial. Specific aims were firstly to describe the different ways in which the intervention was implemented, and secondly to explore the contextual factors moderating the quality of intervention delivery, in order to inform ‘real world’ implementation of the intervention.

**Methods:**

Safewards was implemented on 16 mental health wards in England. We used Research Assistants (RAs) trained in participant observation to record qualitative observational data on the quality of intervention delivery (n = 565 observations). At the end of the trial, two focus groups were conducted with RAs, which were used to develop the coding framework. Data were analysed using thematic analysis.

**Results:**

There was substantial variation in intervention delivery between wards. We observed modifications to the intervention which were both fidelity consistent and inconsistent, and could enhance or dilute the intervention effects. We used these data to develop a typology which describes the different ways in which the intervention was delivered. This typology could be used as a tool to collect qualitative observational data about fidelity during trials. Moderators of Safewards implementation included systemic, interpersonal, and individual factors and patient responses to the intervention.

**Conclusions:**

Our study demonstrates how, with appropriate training in participant observation, RAs can collect high-quality observational data about the quality of intervention delivery during a trial, giving a more complete picture of ‘fidelity’ than measurements of adherence alone.

**Trial registration:**

ISRCTN registry; IRSCTN38001825. Registered 29 August 2012

**Electronic supplementary material:**

The online version of this article (doi:10.1186/s13063-017-2189-8) contains supplementary material, which is available to authorized users.

## Background

### Models of intervention fidelity

Intervention fidelity is defined as the degree to which interventions are implemented as intended by their developers [1]. Understanding intervention fidelity is an essential part of the evaluation of complex interventions not only because it is central to the validity, reliability and so generalisability of trial findings, but also because studies can be used to identify barriers and facilitators to successful implementation, and so provide important information about factors likely to impact the uptake of an effective intervention into routine clinical practice [2]. Fidelity is a complex construct, and there is currently no consensus about what the best indicators of fidelity are and how these should be measured during a trial. Indicators of fidelity included in the literature cover implementation fidelity, concerned with the mechanics of delivery, such as adherence to intervention protocols and dosage or exposure to the intervention, and theoretical fidelity, which examines the extent to which the theoretical constructs of an intervention, or its ‘active ingredients’, are delivered by practitioners [3, 4]. Most studies of fidelity focus on a score determined almost exclusively by adherence; that is, how far those responsible for delivering an intervention actually adhere to the trial protocol [5]. However, implementation science is increasingly recognising the importance of elements of theoretical fidelity, because, for example, an intervention can be delivered to protocol but delivered badly, or can be adapted to fit certain contexts yet still retain the theoretical constructs or ‘essence’ of the intervention [6, 7]. Theoretical fidelity can be assessed by monitoring the way in which an intervention is implemented; that is, the quality of delivery [4]. Approaches to measuring theoretical fidelity include the use of standardised tools which record the extent to which the ‘essential elements’ of an intervention are delivered [3], or frameworks which support differentiation of fidelity-consistent, or fidelity-inconsistent, adaptations to an intervention [8].

It has been argued that to evaluate fidelity properly it is necessary to examine not only the various components of fidelity, but also the factors that affect fidelity, as this can explain how interventions are delivered in the ‘real world’ and why interventions may, or may not, be adopted into routine practice [5]. Carrol and colleagues [5], developed a framework which identifies potential moderators of fidelity, including intervention complexity, participant responsiveness and strategies to facilitate implementation. This model was developed further by Hasson and colleagues [9], who highlighted the importance of ‘contextual factors’ such as staff enthusiasm, organisational routines and resources. These contextual factors are likely to be particularly important when considering the translation of innovative, evidence-based interventions into routine practice. Despite long-standing recognition that mental health wards are complex, often chaotic, environments, few studies have examined implementation of interventions, either in trial conditions or routine practice, within these services.

### Qualitative insights into intervention fidelity

Qualitative process evaluations are now widely accepted as offering valuable data on implementation issues in randomised controlled trials (RCTs) [10–12]. Process evaluations commonly involve collection of data from stakeholders using semi-structured interviews and focus groups. Whilst interviews and focus groups are well suited to gathering data on participants' views and experiences they cannot capture enacted *behavioural manifestations* of intervention delivery. Moreover, data collected in interviews is typically a retrospective reconstruction shaped by various factors whilst qualitative observational studies can allow researchers to track changes in implementation over time [9]. These methods have therefore been identified as being particularly valuable in studies of fidelity [9]. Despite these advantages, most observational data utilised in process evaluations comprise quantitative ratings of adherence (e.g. [13, 14]), whilst structured qualitative observations are rarely used [10] and methods of collecting these data during RCTs are not well described in the literature.

In this article we report on a qualitative process evaluation nested within the Safewards cluster randomised controlled trial, designed to provide insights into intervention fidelity [15]. Our study aimed to use qualitative observational methods to (i) describe the different ways in which the intervention was implemented, and (ii) to explore the contextual factors moderating the quality of intervention delivery, in order to inform ‘real world’ implementation of the intervention.

## Methods

### Description of the Safewards trial and its results

Safewards is a complex intervention designed to reduce conflict and containment on mental health wards; ‘conflict’ is a term used to describe behaviours that put the safety of the ward community at risk, such as physical and verbal aggression, absconding, substance misuse, self-harm and attempted suicide, whilst ‘containment’ is the range of different methods used by staff to control conflict, including administration of sedative medication, increased observation, manual restraint and seclusion [16]. The trial comprised 31 mental health wards at 15 hospitals in nine NHS Trusts within 100 km of central London. Inclusion criteria were acute psychiatric wards for adults of any gender. Wards were excluded if they had a specialist function, had planned major changes, or where two or more of the following criteria were met: no permanent ward manager in post, a locum consultant solely responsible for inpatient care, >30% nursing staff vacancy rate. Three random selections were made: (i) hospitals, (ii) two wards at each hospital, (iii) allocation to experimental or control. Wards were randomly allocated to implement either the Safewards Intervention (‘Safewards’; n = 16 wards) or a comparator intervention designed to promote staff wellbeing so that they could support patients to the best of their ability (n = 15 wards). Simple randomisation was performed in each case by the designated staff member at King’s College Clinical Trials Unit. A total of 564 staff consented to participate.

The trial was grounded in the Safewards model which posits that conflict originates in various domains within and outside of mental health wards and identifies a number of staff actions which can influence the frequency of conflict or containment incidents [17]. The intervention comprised a set of ten practices designed to optimise ward social structure and staff-patient interactions (see Table 1). The intervention was implemented at a cluster level, as implementation was a collective endeavour of the nursing team, and the intervention was delivered across the ward community. The trial was conducted over 24 weeks in three phases (baseline, implementation and outcome), each lasting 8 weeks. Components of the Safewards interventions were introduced in a phased approach during the implementation period. Research Assistants (RAs) collected outcomes data, trained ward staff and supported intervention implementation. RAs attended a 2-week training course covering the Safewards model and interventions, and the trial protocol and procedures before the start of the study. They were provided with resources to support implementation, including an RA handbook, training presentations, handbooks for staff, and intervention materials (freely available from www.safewards.net). The ward manager was asked to identify at least ten members of staff from their team who would act as a ‘Champion’, to lead the implementation of a specific component of the Safewards intervention and provide regular updates to RAs on progress.

**Table 1:** Quality of intervention delivery

Quality of implementation	Definition	Observations (examples are taken from our observational data)
Enhancement	Practitioners build on the intervention (“go the extra mile”) to optimise its effectiveness	[Nurse] who is the champion for Soft Words told me that she had changed the poster for the day and that she had gone around the ward showing it to all the nursing staff
Protocol compliant	Practitioners implement the intervention as instructed	[Nurse] approached me to let me know she did a 1:1 session and her patient was agitated so she had offered him the Calm Down Methods box. She said he chose the herbal tea and penguin to hug overnight
Fidelity-consistent modification	Practitioners adapt the intervention to make it work, or work better, in a particular context, whilst retaining the essential elements of the intervention	After we had spoken to [ward manager] it was agreed that we would amend some of the language in the [Soft Words] posters so that all staff would be able to understand the message and be involved in the intervention
Business-as-usual	Practitioners implement the intervention as instructed but practice is unchanged because they view it as what they were already doing before the trial started	[Ward] did prepare for a restraint but staff were able to contain the situation by asking other patients not to interfere and offering to make the situation better for the patient by going to the shops and buying him the items he was requesting. Staff were working within the Talk Down framework as they generally do and this appeared effective. However, I believe that this was more reflective of how [ward] generally runs than of the intervention itself
Dilution	Participants do not do all they are supposed to do, such that this will dilute the impact of the intervention	Staff came up with a lot of [Mutual Expectations] suggestions however many were not in keeping with the rationale of the intervention – felt like a set of rules for patients other than mutual expectations (e.g. families must call the ward first before visiting, no takeaways after 8 pm etc.) Although almost all staff had been trained in this intervention. I had spoken to the manager about it in detail; I felt that they struggled to understand the values underpinning it
Fidelity-inconsistent modification	Practitioners adapt the intervention in a way that is not in keeping with the ‘spirit’ of the intervention, which would probably reduce or nullify its impact	He told me how staff had discussed using the [Calm Down Methods] iPod as a reward for good behaviour for the disruptive patient on the ward. They also planned to take it away should he cause disruption
No implementation	Practitioners do not implement the intervention	Gave him and two other night staff Know Each Other forms. [Healthcare Assistant] vehemently refused to fill it in saying she doesn’t want them to know anything because they [the patients] will make fun of you

To assess implementation fidelity (adherence), RAs completed a checklist on every ward visit scoring adherence to the implementation protocol (Additional file 1). Fidelity checklist scores were converted into percentage implementation scores.

### Data collection

Two methods of data collection were used during this study. The primary method of data collection was participant observation; observational data were used to develop a typology which described the different ways in which the intervention was implemented (Table 2), and to identify the moderators of intervention delivery. Two focus groups were also conducted with RAs which were used for critical reflection on our typology and to generate new data which were also included in our analysis of the moderators of intervention delivery.

**Table 2:** Safewards interventions^a^

Clear Mutual Expectations	A set of mutual expectations identified by ward staff in partnership with the patient community, for staff, patients and ward visitors, based around values, respect and mutual support, which are displayed on posters around the ward
Soft Words	Short statements outlining potential strategies to use when handling flashpoints (e.g. responding to requests, setting limits, etc.), printed on postcards and a poster hung in the nursing office which is changed every few days
Talk Down	A poster summarising basic to advanced de-escalation techniques placed in the nursing office. One member of staff who is expert in de-escalation spends about 10–15 minutes with members of staff explaining the poster and what it means
Positive Words	When giving handover staff say something positive about what each patient has been doing during the shift, or draw attention to some positive quality they have, or something positive about the way in which staff supported them
Bad News Mitigation	Staff maintain an awareness of occasions and events that might cause people to feel upset or angry (e.g. phone calls or family, unwelcome news from the treatment team). The staff work with the team to express the bad news sympathetically, or support the person after it has happened
Know Each Other	Staff and patients produce a profile of who they are as a person (e.g. hobbies, interests, likes and dislikes, etc.) which is made available to everyone via either a notice board or a folder on the ward
Mutual Help Meeting	A voluntary meeting of all patients and staff on duty, to be held preferably in the morning, about how everyone can help everyone else during the rest of the day. The meeting follows a structured agenda (rounds of thanks, news, suggestions, requests and offers) stressing mutual support agreements. The meeting does not have to be chaired by a staff member
Calm Down Methods	A box of distraction, sensory modulation and relaxation tools to offer to people when they appear to be upset, tense or agitated (e.g. stress toys, mp3 players with soothing music, light displays, textured blankets, etc.)
Reassurance	Explanations and reassurance offered to all patients following potentially frightening incidents
Discharge Messages	A display of positive messages about the ward from people who have been discharged, covering what they liked about the wards and a helpful piece of advice for new patients

^a^Full descriptions of these interventions coupled with training videos are freely available online at www.safewards.net

Qualitative observational data were collected during the implementation and outcome phases of the trial by 11 RAs. RAs visited each ward two or three times each week, typically spending 2–6 hours on the ward. They were aged between 21 and 40, and nine were women. All held degrees in a social science or mental health nursing and had worked on acute mental health wards in a range of roles including as a Healthcare Assistant (n = 6), Mental Health Nurse (n = 2) and Assistant Psychologist (n = 3).

RAs were asked to intentionally observe and record, immediately after each visit, the ‘most notable response’ of nursing staff to (a) Safewards as a whole, and (b) a specific intervention component (i.e. Soft Words, Talk Down etc. - see Table 1). Because an aim of this study was to describe the variety of different ways in which the intervention was implemented, we documented the ‘most notable response’ rather than an overall response. This maximised our chances of capturing the full range of responses to implementation, because, for example, asking RAs to record an overall response was likely to return many descriptions of ‘protocol-compliant’ implementation, and few, if any, of the more extreme responses, such as non-implementation or enhancement (Table 2). It could also be difficult to gauge the ‘overall response’ of a ward where there may be a range of different responses from individual members of staff implementing an intervention. RAs were asked to record what the staff response meant to them and why, i.e. to explain why it was considered to be a notable response, and were given examples in training and written guidance (see below) as to what these might be, for example a positive response have felt like an important ‘breakthrough’, while a negative response may ‘concerning’ because it indicates a lack of trust. Field notes for each observation were recorded using a structured data collection sheet which captured an account of the following:

- context
- what happened, including: what was said/done that led the RA to infer a ‘response’ of some sort; who was involved (i.e. which members of staff), and how the RA responded at the time (i.e. the interaction between staff and RA)
- implications for intervention implementation

To promote consistency in type and quality of data collected all RAs participated in a half-day training session led by an experienced ethnographer (AQ) and the Trial Co-ordinator who had collected data for the earlier Safewards pilot study (KJ), and were provided with written guidance regarding the practice of participant observation and how to record good-quality notes. All data collected were reviewed after 2 weeks by AQ and KJ with detailed feedback and further guidance provided to RAs individually as appropriate. Observational data sheets, completed by hand at the time of the observation, were transcribed into electronic form and uploaded to NVivo for thematic analysis.

At the end of the trial, all RAs participated in one of two, 2-hour focus groups facilitated by AQ and KJ. During each focus group RAs were presented with a draft typology of implementation quality (Table 2) and were asked to provide feedback. Factors identified in the data as influencing intervention delivery were explored further to generate new data and rich descriptions of the possible moderators of implementation quality. Focus groups were audio recorded and transcribed for analysis.

### Data analysis

We conducted an inductive thematic analysis of observational and focus group data as follows:

#### Development of typology

Observational data were coded for intervention implementation to one of three categories; take-up, adaptation and non-implementation. The data coded to each theme were further interrogated to develop a typology describing the quality of intervention implementation during the trial (Table 2). To test the reliability of the coding frame a random sample of 20 data sheets were independently coded by KJ and AQ. There was 83% agreement between coders indicating a reliable framework.

#### Analysis of the moderators of implementation delivery

A coding frame identifying the contextual factors which influenced the quality of delivery was developed through an initial analysis of the observational data. This was explored further in the focus group and the coding frame was then applied to focus group data. Codes were reviewed and refined through an iterative process involving regular meetings between KJ and AQ.

## Results

A total of 565 observational data sheets were completed across the 16 wards implementing the Safewards intervention over a 16-week period covering the implementation and outcome phases of the trial (mean = 2.2 structured observations per week per ward). The mean fidelity to the Safewards intervention by ward was 38% (SD 8, range 27–54%, n = 271) and for the control intervention was 90% (SD 9, range 69–99%, n = 209). There was no association between adherence scores and outcomes; however, Safewards was associated with a significant reduction in frequency of both conflict and containment [15].

Here the results of the qualitative observational study are presented in two parts: first, we outline a typology of implementation quality for the Safewards RCT, and second, describe moderators of the quality of implementation of Safewards observed by RAs during the trial.

### Quality of intervention implementation: a typology

The first aim of this study was to provide insights into intervention fidelity during the trial by describing the different ways in which the intervention was implemented; these are outlined in Table 2. Table 2 presents a typology of implementation quality, in which different forms of implementation are differentiated by the extent to which the delivery of the intervention adhered to the logic and hypothesis underpinning its design. This captures a range of responses from ‘enhancement’ of the intervention, where staff go above and beyond the implementation instructions and make a special effort to build on the intervention, to cases where the intervention is not implemented at all (‘no implementation’). Between these extremes, and in line with current conceptualisations of fidelity, we found that staff made modifications to the intervention which could be described as fidelity consistent (i.e. changes to make it ‘work’ in a particular context) or inconsistent (i.e. changes which would reduce its impact) [8]. Whilst on some wards practitioners added components to the intervention which were likely to enhance its effectiveness (‘enhancement’), we also observed cases where staff neglected to implement essential intervention components, which was likely to dilute its effect (‘dilution’). We found that for some intervention components practice on wards adhering to the protocol was unchanged (business-as-usual) as these wards had been implementing a similar approach before the trial.

### Factors moderating implementation quality

The second aim of this study was to describe the factors moderating the quality of intervention delivery to inform effective ‘real world’ implementation of the Safewards intervention. We found the following factors were critical in shaping the ways in which the various components of Safewards were delivered;

- the ward environment and organisation
- team culture and dynamics
- staff skills, confidence and understanding
- staff values and beliefs about the Safewards intervention and trial
- patients’ responses to the intervention

We describe each of these below, illustrating with examples from observational data recorded by RAs after each ward visit (denoted “Obs, Ward ID, Researcher ID”) and comments made by them in focus groups (denoted “FG, Researcher ID”). At the end of this section we give examples of how observational data, captured at regular intervals throughout the trial, could be used to explain changes in fidelity over time.

#### The ward environment and organisation

The ward environment was a major determinant of implementation quality; a busy or chaotic ward, often attributed to staff shortages, unwell patients and ward incidents, was frequently described as a barrier to implementation. The broader organisational context was also important, for example upcoming inspections by the Care Quality Commission (the independent health services regulator in England), or discussions at a senior level about ward closures influenced intervention delivery. These characteristics were seen to have a major impact on nurses’ capacity, or motivation, to change current practice:

*Staff are responding well to the project in general, but are under staffing pressures and have a number of difficult patients – making them slower to take up the interventions. (Obs, W04, RA7)*

*There’s a lot of pressure and expectation from people outside of the wards. Not only about the study but about how they work in general…which for some people led to them trying their best but feeling quite burnt out. But for others it was more about them kind of taking control by not doing it. (FG, RA1)*

The daily routines of the ward and its function were also influential. For example, whilst some teams diluted, or did not implement interventions that were seen to be at odds with ward structures, others adapted them to ‘fit’ with current practice:

*They couldn’t have a Discharge [Messages] tree on the ward…So they [ward staff] said we’ll make one, they made it and they also said ‘because we’re an assessment ward, it doesn’t make sense to discharge them because maybe only one in ten is discharged’. So then it was [renamed] a positive word tree… so any patient wanting to write something nice or positive it could go on the tree. (FG, RA2)*

#### Team culture and dynamics

Alongside differing organisational contexts, RAs observed strong ward cultures, or identities, which influenced the ways in which teams responded to the intervention:

*That’s one of the attitudes that I found on the ward with being, I called it cool kid syndrome, where they just wanted to be seen as being like, good but not bothered, kind of blasé about stuff. (FG, RA3)*

*They seem to have a very strong identity… a very strong ward, with a, ‘they know what they’re doing’ attitude or they think they know what they’re doing and if they don’t like what I was suggesting then it was just ‘no’. If they did, I didn’t need to say anymore they got the staff handbook out, read it, and it was done.* (*FG, RA1*)

The dynamics within some teams meant that the responses of individual staff were, in some cases, largely determined by their colleagues’ reactions to the intervention:

*I had one HCA [Healthcare Assistant] who absolutely loves the Know Each Other [intervention] but she took it out of the folder and I asked her why she’d done that, and she said that one of the other HCAs went to her and said ‘you shouldn’t have put so much information there, you’re going to offend the patients’… And it just made her really, really unsure about herself and her responses and even paranoid a little bit as well. (FG, RA4)*

Leadership was also important; teams which had no ward manager for a period during the study, or where there was poor leadership, were less keen to adopt the intervention, and the support of a ward manager, or its absence, could either legitimise or undermine implementation:

*He [the ward manager] was quite disconnected from the group in general… so he didn’t really have any, kind of, authority. So you couldn’t really go to him to get support for anything… he’d say something, [but] that wouldn’t be effective because, you know, that that’s actually going to make them less likely to do it.* (*FG, RA8*)

*[spoke with ward manager] about the Know Each Other intervention. She stated that she would bring it up at handover tomorrow and speak to any staff that she saw today. I asked her if she could think of any reason that staff would be reluctant to complete one; she said no – and said if they were told to complete one then they would… I left the conversation feeling positive that [ward manager] would speak to staff about the intervention as [ward manager] usually followed through on her words. (Obs, W05)*

#### Staff skills, confidence and understanding

The skills and experience of staff were identified as critical determinants of implementation, for example, in some cases a lack of confidence, or understanding meant that intervention components were not implemented or were diluted. This was a particular issue on wards with large numbers of bank (temporary) staff, who had not attended Safewards training, or where interventions which required a level of clinical skill, such as the Mutual Help Meetings, were led by inexperienced Healthcare Assistants without the support of trained clinicians. Some staff also struggled to understand the function or theory underpinning components of Safewards, and this often led to dilution or negative adaptation of the intervention:

*The Mutual Help Meeting under the banner of daily planning meeting had occurred everyday this week although the round of news section looks to encompass world news rather than ward news as evidenced by some minutes about the Boston bombers in the log book*. (*Obs, W28, RA6*)

In other cases, RAs felt that teams had the necessary skills, but observed a lack of confidence amongst some staff to try new ways of working:

*Based on what I saw, each ward would have a fair portion of those people [nurses] that lack the confidence. ‘Cause there was some on [named ward] that were fairly gung ho and ready for anything, didn’t have an demonstrable fear about holding these meetings, and then some people you can just see their reluctance coming from them. (FG, RA3)*

#### Staff values and beliefs about the Safewards intervention and trial

Effective implementation was determined not only by staff skills and understanding, but also whether or not the values underpinning Safewards fit with personal beliefs about nursing practice and predictions about the potential impact of the intervention. Views about research were also influential; RAs reported that a minority of clinicians were openly critical of research and, because Safewards was a research study, questioned the grounds for the interventions. Some staff considered the RAs responsible for implementation, in part due to a perceived lack of ownership:

*[Some nurses had a] passive sort of attitude towards the whole thing, that ‘oh your here, you’re doing this, it’s your project’.* (*FG, RA4*)

*A band 6 [nurse] on my ward had a thing against research, in general - that research never changes anything, that they’ve had lots of experiences of various different things on the ward that just makes no difference. (FG, RA5)*

What were described by some RAs as ‘core beliefs’ of staff were considered a key influence on the likelihood of their engagement in Safewards. For example, staff had divergent views about the issue of power sharing with patients and the perceived risks involved:

*Safewards isn’t authoritarian at all and people coming from that kind of dialect and that way of working, I think that they would have seen it as something very alien to them and something with dangerously few barriers or boundaries… And I talked about this with one of the youngest staff who was more eager to do the project. She said that a lot of the ward is held back by the old school nurses that are still coming from this different philosophy of nursing. (FG, RA3)*

*Whilst on the ward I spoke to the Know Each Other champion who reported the intervention wasn’t going well. He reported numerous staff members had refused as they felt unwilling to share information about themselves with service users. (Obs, W7, RA9).*

Staff were more likely to adopt or “go the extra mile” to enhance an intervention if they thought it would lead to positive outcomes. They were also more likely to implement it if they believed it would develop current practice. For example, in some cases a Safewards intervention was not taken up because it was seen to replicate what the ward was already doing, or because current practice was viewed as superior:

*[Nurse] started to offer the [Calm Down Methods] box to various patients, and also expressed to [staff names] how much he thought this would help in difficult situations. [Nurse] then sent an email to all staff members describing the intervention and encouraging them to use it. (Obs, W21, RA5)*

*They had a system for that [Bad News Mitigation] already in place and to them it was a common sense thing, they manage upcoming bad news that they know about sensitively. They stuck to their guns on that one quite a lot which is why they rejected that intervention quite a lot. (FG, RA3)*

#### Patients’ responses to the intervention

RAs found that feedback from patients on the ward influenced adoption of a practice, with ‘positive’ responses encouraging implementation:

*The patients went to the staff and gave them really good feedback about it, which made the staff actually do it for the following week. So in that way I guess they were empowered by positive feedback from the patients.* (*FG, RA3*)

Whilst negative could lead staff to abandon new practices:

*“I think they started to abandon some of the some of the interventions because they weren’t seeing an immediate effect from it. The Discharge Messages tree, which was probably the best received one, was attacked by a patient twice in a week and then never really recovered from that*. (*FG, RA6*)

#### Observations of change in implementation over time

Observational data, captured at regular intervals throughout the trial, could be used to explain changes in fidelity over time. During the study, events such as major ward incidents, changes ward staffing or changes to the function of the ward had a significant impact on implementation quality:

*Today I spoke to the new ward manager, [she] apologised to me saying “I’m sorry I haven’t had much time to focus on Safewards as I am trying to get to know everything on the ward.” [Ward Manager] also stated that it is “a lot to understand and I wish I started from the beginning of the project.” I feel that [she] was politely saying that she feels like is it too late for her to focus fully on the project. [Obs, 21]*

And on a number of wards RAs found that staff became less motivated towards the end of the study, which meant they stopped implementing components of the intervention:

*“So things like the calm down box on [ward], near the end it wasn’t even being used, it wasn’t being offered to people, and, umm, I think in the last month, so it started quite a while before actually, they’d only been bringing it out once a week, so the patients could take things from it, but then close to the end of the study they’d stop doing that as well. Umm, things like the discharge tree, people weren’t being asked to fill in messages even though there were patients being discharged. (JD, FG)*

## Discussion

We used participant observation methods to generate qualitative data about the quality of delivery of the Safewards intervention during the Safewards randomised controlled trial (RCT). These data were used to develop a typology which describes the different ways in which an intervention can be delivered. We identified a diverse range of contextual moderators of the quality of Safewards implementation, including the ward environment, individual staff and team characteristics and patient responses to the intervention.

Qualitative observational methods have been identified as being particularly valuable in studies of implementation and fidelity, however are rarely used as the primary method of data collection in such studies [9]. Our study demonstrates how, with appropriate training in participant observation, RAs can collect rich observational data about fidelity during a trial. Such methods can be particularly useful in studies of implementation during a cluster RCT, where implementation is at group rather than individual level. For example, we identified interpersonal moderators of implementation such as team cultures and identities, leadership and the influence of peers, which were unlikely to have been elicited through interviews or focus groups with ward staff.

Traditionally studies of fidelity have focused on adherence, however it is increasingly recognised that to give a complete picture of fidelity and its relationship to outcomes, studies must take a multi-dimensional approach and measure elements of theoretical fidelity, such as the quality of implementation. This is because interventions are often modified by clinicians to work within a particular context, and so demonstrate low adherence to the implementation protocol, yet may still be congruent with intervention theory [3]. Our typology builds on existing approaches to monitoring the quality of implementation, or theoretical fidelity (e.g. [3, 8]), and provides a nuanced description of the extent to which quality of implementation reflects the theory of an intervention, and its likely impact on intended outcomes. Observational data such as this could also be used to support effective ‘real world’ delivery of evidence-based interventions by providing examples of where ‘dilution’ can occur, or how an intervention could be effectively adapted to fit within a particular context. We believe this typology could be applied in similar studies alongside more traditional measures of adherence, such as those used during the Safewards trial (Additional file 1), allowing trialists to better account for the relationships between outcomes and fidelity. For example, low adherence was reported during the Safewards trial and there was no association between adherence scores and outcomes, however there was a significant reduction in conflict and containment following implementation of the intervention [15], leading some commentators to question whether it was Safewards that made the difference ([18]; see [19] for response). Our process evaluation found examples of where staff responded such that effectiveness would be optimised (enhancement) but where a low fidelity (adherence) score would have been recorded and also the converse, e.g. where a Mutual Help Meeting (Table 1) was recorded as happening every day in the structure specified in the protocol, but where it had been adapted in a way that was not in keeping with the spirit of the intervention.

The most commonly used framework for the study of fidelity is the Conceptual Framework for Intervention Fidelity developed by Carrol et al. [5]. Our findings support the addition of ‘context’ as a key moderator of fidelity [9], although what is meant by ‘context’ in relation to Carroll’s [5] framework is currently unclear. In line with previous studies of implementation, and the diffusion of innovation [20, 21], we found a range of individual, group and systemic factors to influence the quality of intervention delivery. These ‘contextual factors’ are particularly important in studies of fidelity as these data provide information about the barriers and facilitators to implementation likely to occur in routine clinical practice. These are likely to vary significantly between studies, and our typology provides a structured framework which can be used to capture these data during a trial.

Since completion of the trial Safewards has been implemented in Australia, Canada, Germany, Denmark, and Finland. Social media platforms are being used to share learning, including an active implementation discussion group with over 5000 members. Some wards have chosen to use the trial adherence questionnaire in local monitoring - we would encourage them to also consider recording observations about the quality of intervention delivery (e.g. Table 2). Our findings indicate that successful implementation of Safewards requires strong leadership and ‘buy in’ from the majority of ward staff. Implementation strategies initiated at an organisational level, without the support of ward staff, are likely to experience significant challenges, as is implementation on wards where there may be structural instability, such as understaffing, large numbers of temporary staff, temporary or absent ward manager or other major tasks or initiatives, such as upcoming inspections. This is supported by a recent evaluation of Safewards that reported poor adherence linked to operational demands and attitudinal barriers amongst staff, and concluded that staff should have been better prepared for implementation [22].

### Limitations

The data we gathered for the process evaluation do not allow us to investigate the association between fidelity and types of staff response because we only elicited a partial picture; that is, the “most notable” response of nursing staff recorded during each site visit. RAs were instructed to give concrete examples to support their observations, however, the data may be subject to observer bias. Whilst participant observation has a number of strengths, we did not directly seek the views of staff through formal interviews or focus groups.

## Conclusions

Our study demonstrates how, with appropriate training in participant observation, RAs can collect high-quality observational data about the quality of intervention delivery during a trial, giving a more complete picture of ‘fidelity’ than measurements of adherence alone. We identified a diverse range of moderators of the quality of Safewards implementation, including the ward environment, individual staff and team characteristics and patient responses. These data could be used to inform ‘real world’ implementation of the intervention.

## Additional file

Additional file 1: Researcher Visit Fidelity Checklist. (PDF 24 kb)

## Abbreviations

RA: Research assistant
RCT: Randomised controlled trial
