# Supplementary material for: Quality of intervention delivery in a cluster randomised controlled trial: a qualitative observational study with lessons for fidelity
Source: Trials. 2017 Nov 17;18:548. doi: 10.1186/s13063-017-2189-8 (PMC5693543; doi:10.1186/s13063-017-2189-8)
Supplement: Additional file 1: — Researcher Visit Fidelity Checklist. (PDF 24 kb) [file 13063_2017_2189_MOESM1_ESM.pdf]

Safewards

Researcher Visit Fidelity Checklist: Organisational Ward

Please complete one of these checklists each time you visit the ward during the implementation and outcome periods

A1 Is there at least one Mutual Expectations poster on the wall publicly?

Yes .... ☐
No .... ☐

A2 Is the 'Talk Down' poster displayed on a wall visible to ward staff?

Yes .... ☐
No .... ☐

A3 Is the 'Soft Words' poster displayed in the staff office?

Yes .... ☐
No .... ☐

A4 Are the Discharge Messages visible to service users?

Yes .... ☐
No .... ☐

A5 Number of Discharge Messages visible?

	1	*2*	*3*	*4*	*5*	*6*	*7*	*8*	*9*	*10/>*
Number of discharge messages visible	☐	☐	☐	☐	☐	☐	☐	☐	☐	☐

A6 Is the 'Know Each Other' folder available in a public area of the ward?

Yes .... ☐
No .... ☐

A7 Number of Know Each Other profiles in the folder?

	1	*2*	*3*	*4*	*5*	*6*	*7*	*8*	*9*	*10/>*
Staff	☐	☐	☐	☐	☐	☐	☐	☐	☐	☐
Service users	☐	☐	☐	☐	☐	☐	☐	☐	☐	☐

A8 Check the Calm Down Methods log book and indicate the number of Calm Down Methods used since your last visit.

	1	*2*	*3*	*4*	*5*	*6*	*7*	*8*	*9*	*10/>*
Number of Calm Down Methods used	☐	☐	☐	☐	☐	☐	☐	☐	☐	☐

A9 Check the recent PCCs for incidents of physical aggression, attempted suicide, seclusion, restraint, coerced IM medication or transfer to PICU, then indicate all answers that apply below

There were no such incidents since the last visit .... ☐
There were one or more of such incidents .... ☐
The staff indicate that Reasurrance took place (in at least one case, if more than once such incident occurred)........ ☐

A10 Check the Mutual Help meeting log book, and indicate the number of meetings that took place since your last visit

	1	*2*	*3*	*4*	*5*	*6*	*7*	*8*	*9*	*10/>*
Number of mutual help meetings logged	☐	☐	☐	☐	☐	☐	☐	☐	☐	☐

A11 Number of handover checklists complete since the last visit

	1	*2*	*3*	*4*	*5*	*6*	*7*	*8*	*9*	*10/>*
Number of handover checklists completed	☐	☐	☐	☐	☐	☐	☐	☐	☐	☐

A12 **Date**

A13 **Ward Research number**

	0	*1*	*2*	*3*	*4*	*5*	*6*	*7*	*8*	*9*
First digit	☐	☐	☐	☐	☐	☐	☐	☐	☐	☐
Second digit	☐	☐	☐	☐	☐	☐	☐	☐	☐	☐

A14 **Research Assistant number**

	0	*1*	*2*	*3*	*4*	*5*	*6*	*7*	*8*	*9*
First digit	☐	☐	☐	☐	☐	☐	☐	☐	☐	☐
Second digit	☐	☐	☐	☐	☐	☐	☐	☐	☐	☐

Ward research number

RA Initials

Date

One of these sheets needs to be completed after every ward visit. Please fill in the two boxes below. Refer to the Guidance Notes as required.

B4 **How are staff currently responding to the trial as a whole? Please record the most notable response you encountered during this visit (continue overleaf if necessary).**

B5 **How are staff currently responding to particular Safewards interventions? Please record the most notable response to a particular intervention that you encountered during this visit (continue overleaf if necessary).**

B4 Continued

B5 Continued
